# Understanding cognitive dysfunction in depression: perspectives and practices of UK health and social care professionals, a qualitative study

**DOI:** 10.1136/bmjopen-2025-109285

**Published:** 2026-02-12

**Authors:** Amirah Akhtar, Emmanuel Nwofe, Shabana Shafiq, Sahdia Parveen, Karen Windle

**Affiliations:** 1 Department of Applied Dementia Studies, University of Bradford, Bradford, UK; 2 School of Nursing and Paramedic Science, Ulster University, Coleraine, UK

**Keywords:** Dementia, Depression & mood disorders, Cognitive dysfunction, QUALITATIVE RESEARCH, Primary Prevention

## Abstract

**Abstract:**

**Objective:**

To explore how health and social care professionals (HSCPs) in the UK conceptualise and respond to cognitive dysfunction in depression, including its potential long-term implications for brain health and dementia risk.

**Design:**

A qualitative, semi-structured interview study. Data was analysed using a code-book approach to thematic analysis.

**Setting and participants:**

The study was conducted in the UK, with HSCPs from diverse professional backgrounds including general practitioner, psychology, psychiatry, mental health nursing, psychological well-being practitioner and occupational therapy. A total of 12 participants were recruited via purposive and convenience sampling.

**Results:**

Three master themes were developed, (1) Cognitive dysfunction in depression, (2) Persistence of cognitive dysfunction and (3) Depression and dementia risk. HSCPs expressed challenges in screening for cognitive dysfunction in depression, particularly as dementia-related screening tools were used which may not be sensitive enough to detect depression-related cognitive deficits. A number of potential explanations were reported as to why cognitive dysfunction may persist after mood symptoms have lifted. These included substance misuse, role of education, neurological conditions and depression as a prodrome to dementia. Depression as a potential risk factor for poorer brain health in the context of dementia risk reduction was not communicated in clinical settings to service users. Barriers to communication included lack of evidence base on depression as a potential risk factor, as well as lack of guidance on communication practices in the context of mental health issues.

**Conclusions:**

Cognitive dysfunction in depression is a complex phenomenon and remains under-explored. Challenges around identification and screening indicate a need for validation studies of cognitive screening measures for use in mood disorders, as well as pilot, acceptability and feasibility trials of interventions targeting cognitive functioning in mood disorders. Mixed-methods research is warranted to understand whether guidance on communicating depression as a risk factor for brain health is required and/or justified.

**STRENGTHS AND LIMITATIONS OF THIS STUDY**

- Diversity of professional backgrounds represented to gain insights.
- Adding to the limited literature on depression and dementia risk reduction.
- Purposive/convenience sampling approach may limit generalisability of the findings.
- Some professional backgrounds are over-represented in the sample.

## Introduction

 Cognitive dysfunction in major depressive disorder (MDD) involves impairments across various domains, including attention, verbal and non-verbal learning, short-term and working memory, visual and auditory processing, problem-solving, processing speed and motor skills. It is often considered a key contributor to the functional impairments observed in individuals with MDD.^1^ In recent years, several studies have demonstrated the impact of cognitive dysfunction during depression on the individual’s well-being and quality of life. Akhtar *et al*^2^ conducted a qualitative study exploring subjective cognitive symptoms in a British South Asian sample, with recurrent depression. The findings indicated that difficulties in attention and concentration impacted psychosocial functioning, increasing the risk of social isolation and loneliness, a risk factor for long term conditions such as dementia.^3^ Participants also reported difficulties learning new information, which negatively impacted their occupational functioning. Retaining information such as remembering prayers was also a challenge impacting spiritual well-being and overall quality of life. Crowe *et al*^4^ explored the lived experience of subjective cognitive symptoms in participants living in New Zealand. Participants described difficulties completing simple tasks as they reported ‘feeling stuck’ mentally and physically. Rumination and zoning out during conversation were also reported, which negatively impacted psychosocial functioning. These studies demonstrate the burden and complexity of cognitive dysfunction in depression and how it impacts several areas of an individual’s life.

In recent years, there has been growing interest in the long-term neurological implications of depression, particularly its association with increased dementia risk.^3^ The relationship between depression and dementia is complicated as depression is sometimes considered a prodrome to dementia.^5^ Lee *et al*^6^ found that having depression in both adulthood and older adulthood was associated with higher dementia risk. Depression in midlife has been reported as a potentially modifiable risk factor for dementia.^3^ Yet, how health and social care professionals (HSCPs) understand and navigate this link in practice remains under-explored. One of the factors that has been shown to contribute to the recurrence of depression is the presence and persistence of cognitive dysfunction.^7^ Subjective cognitive symptoms have been found to be prevalent and present approximately 85%–94% of the time during a depressive episode and 39%–44% during remission.^8^

It will be understood that much of the literature has focused on subjective cognitive symptoms in depression, as opposed to objective cognitive dysfunction. These constructs are only modestly related and, in many studies, show little or no correlation. Subjective cognitive symptoms often track more closely with affective symptoms, low self-esteem, low self-efficacy and expectancy effects rather than with performance-based cognitive deficits.^9 10^ However, research has indicated that the presence of subjective cognitive symptoms may increase the risk of objective cognitive dysfunction. Wang *et al*^9^ conducted a systematic review and meta-analysis examining the association between cognitive symptoms and risk of cognitive impairment and dementia. Cognitive symptoms were found to be associated with increased risk of objective cognitive dysfunction, including cognitive impairment and incident dementia. A meta-analysis of longitudinal studies found that cognitive symptoms approximately doubled the risk of developing mild cognitive impairment and dementia.^10^ These studies indicate that subjectively reported cognitive symptoms deserve more attention, particularly in the context of depression which has been reported as a potentially modifiable risk factor for dementia.^3^ Although it is important to highlight that many people with cognitive symptoms do not go on to experience objective cognitive disorders. Nevertheless, cognitive symptoms associated with depression could serve as a potential target for early intervention.

A survey of 100 clinicians based in the UK (general practitioners (GPs) and psychiatrists) examined understanding of cognitive dysfunction in major depression. The results suggested that clinicians lacked awareness of cognitive dysfunction in depression, including how to detect, manage and why it may persist after symptomatic recovery.^11^ However, little is known about lived clinical experiences of Health and Social Care Professionals (HSCPs) working in mental health settings and how they conceptualise cognitive dysfunction in depression, including risk communication related to long-term brain health. The term ‘brain health’ is being introduced by dementia charities as a way to transform how people think about dementia risk reduction. Alzheimer Research UK (2021) highlights that the term ‘brain health’ resonates with people of all ages, as opposed to the term dementia which is often associated with the elderly and late life. The term brain health is also less stigmatising than ‘preventing dementia’. Using the term brain health may therefore lead to behaviour change, rather than the concept of dementia risk reduction. Brain health is a term that has had limited use in the UK, despite its growing use internationally.^12^ Given these gaps, there is a pressing need to explore how HSCPs define, assess and respond to cognitive dysfunction within depression, and how, if at all, they consider its implications for future dementia risk.

### Aim

To explore how HSCPs in the UK conceptualise and respond to cognitive dysfunction in depression, including its potential long-term implications for brain health and dementia risk.

## Research questions

- How do HSCPs define and understand cognitive dysfunction in the context of depression?
- What factors do HSCPs believe contribute to the persistence or severity of cognitive dysfunction?
- How do HSCPs identify and assess cognitive dysfunction in clinical practice?
- To what extent do HSCPs associate cognitive dysfunction or depression with dementia risk, and how is this risk communicated to service users?

## Materials and methods

This qualitative, descriptive study used semi-structured interviews to explore knowledge and understanding of cognitive dysfunction during depression. This study is underpinned by a critical realist philosophical position, which assumes that while subjective and objective cognitive dysfunction in depression and depression as a dementia risk factor have been demonstrated to exist as real-world phenomena, professionals’ understandings of these issues are shaped by their social, organisational and professional contexts. The authors assert that all procedures contributing to this work comply with the ethical standards of the relevant national and institutional committees on human experimentation and with the Helsinki Declaration of 1975, as revised in 2013. All procedures involving human subjects/patients were approved by the Chair of the Humanities, Social and Health Sciences Research Ethics Panel at the University of Bradford, reference number E1150.

### Participant recruitment

Participants were recruited via purposive, convenience and snowball sampling across the UK in rural and urban settings. Inclusion criteria included being a HSCP with a minimum of 12 months experience of working with people with depression, in primary (General Practitioner), secondary (Psychiatrist), tertiary health (Neurologist) care and/or community mental health settings (Community mental health nurse). Participants were recruited using existing networks of the research team (face to face) and social media (online). Authors 1 and 2 have expertise in the field of mental health and advertised the study at mental health events where health professionals were present, including psychologists and psychiatrists. Author 5 has expertise in ageing and dementia research and similarly advertised the study at a research networking event attended by academics and clinicians including GPs. Individuals who took part in the study also advertised the project across their teams, resulting in some participants being recruited via snowball sampling. Participants were not previously known to Author 1 prior to study commencement. Participants who expressed an interest in taking part contacted the researchers, and a study information pack was sent. A total of 12 participants were included (see table 1 for participant characteristics). Prior to data collection, all participants provided written informed consent to take part in the study and their anonymised data to be published.

**Table 1:** Participant characteristics

Participant	Gender	Job title	Ethnicity
1	Female	Cognitive Behaviour Therapist	South Asian
2	Female	Clinical Psychologist in Older people’s services	White German
3	Female	Clinical Psychologist	White British
4	Male	Psychologist	South Asian
5	Female	Psychotherapist	White British
6	Female	Neurologist	South Asian
7	Male	Occupational therapist	South Asian
8	Female	General Practitioner	Mixed White and Black Caribbean
9	Female	Psychiatrist	South Asian
10	Male	Senior Psychological well-being Practitioner	South Asian
11	Male	Mental Health Nurse Therapist	White British
12	Male	Junior Doctor	Arab

### Data collection

Interviews followed a topic guide, and questions were developed based on existing literature and discussions across the research team. All interviews were conducted by the first author (female, PhD) via Microsoft Teams. Participants were made aware that the first author was on a dementia capacity building fellowship under the theme of dementia prevention and has an interest and background in mental health research, hence the focus on depression. Only the interviewer (Author 1) and the research participant were present during interviews. Data was collected until saturation was reached. Saturation was conceptualised as meaning or theoretical saturation, in line with thematic analysis, referring to the point at which further interviews did not contribute new insights relevant to the research questions. Although the sample was professionally diverse (including medical, psychological, nursing, occupational therapy and social work perspectives), analysis demonstrated that later interviews increasingly reiterated concepts identified earlier in the process. No new codes were added to the codebook during the final interviews, and the coding framework had reached stability, with existing themes sufficiently capturing the variation across professional roles. This indicated that additional data were unlikely to meaningfully alter or expand the thematic structure, suggesting that saturation had been achieved.

All interviews were audio-recorded and transcribed verbatim. Transcripts were checked for accuracy and anonymised by the first author before analysis. All interviews lasted between 50–60 min, and the interviews were conducted between March and September 2024. Questions explored participants’ knowledge and understanding of cognitive dysfunction in depression, including possible causes, screening and implications for dementia risk. The interview schedule is available in online supplemental file 1. Participants also completed a demographics form. It is important to declare research characteristic and positionality for readers to ensure transparency and credibility. As such, a brief description of the first author follows as they led data collection and analysis.

### Reflexivity

I am a female, Pakistani-Muslim academic research fellow with an interest in mental health and dementia risk reduction. I have conducted qualitative research in depression, subjective cognitive functioning and dementia prevention in minority ethnic communities. I began this work with an appreciation for the complexity of depression and dementia risk. Although I recognise that causality in this area is neither linear nor deterministic, I hold a belief that recurrent depressive episodes can contribute to subjective cognitive dysfunction and overall poorer brain health. In my role as lead researcher, I collected all data, conducting interviews with a wide range of HSCPs (see table 1). I attempted to bracket my assumptions around the presence of cognitive dysfunction in depression and potential dementia risk. For example, bracketing occurred when participants were asked their views on whether or not cognitive dysfunction in depression increased the risk of developing dementia or contributed towards poorer brain health later in life. Regular meeting with co-authors provided an additional layer of critical scrutiny, enabling me to interrogate whether emerging codes and themes were grounded in participants’ accounts or shaped by my prior expectations. During interviews, I ensured questions were open, with non-leading prompts to allow participants to frame their own narratives. Field notes were recorded during and after the interview and discussed with co-authors to ensure interviews are capturing information relevant to answering the research questions. The team included three further female academics and one male. All work within the field of dementia prevention and are knowledgeable of the different dementia risk factors.

### Data analysis

Data was analysed using Braun and Clarke’s approach to codebook thematic analysis. A deductive approach was taken, guided by the research questions to develop a codebook shaped by predefined areas of interest such as persistence of cognitive dysfunction after depression symptoms have lifted and views on depression as a risk factor for dementia. The analysis was conducted at a semantic level, focusing on the explicit, surface meaning of participants’ accounts. Three interview transcripts were double coded by Authors 1 and 2 to ensure consistency and reliability among the coding process. Once this was established, independent coding of transcripts was resumed. Transcripts were coded in NVivo V.12 Pro software.^13^ Following this independent coding and further conversations with co-authors, codes were refined and (where necessary) collapsed.

### Patient and public involvement (PPI)

Individuals with experience of cognitive symptoms in depression were involved in the development of this study. This was part of a wider programme of work looking into depression-related cognitive symptoms in minority ethnic populations. The PPI group reviewed drafts of the topic guide and provided feedback on content, structure and whether the questions covered were meaningful and clear. Their contributions resulted in the inclusion of questions on the identification of cognitive dysfunction. The group felt it was important to ask about support or treatment relating to ongoing cognitive dysfunction, as members had experienced depression multiple times across life. Members of the PPI reported encounters with a diverse set of health professionals during their treatment of depression, some of which included GPs, occupational therapists, psychologists and counsellors. Members were therefore asked whether it would be useful to discuss this topic with a diverse set of HSCPs, which they agreed on. This shaped the participant sampling of the current project.

## Results

The following themes were developed to capture HSCPs’ views and understanding of cognitive dysfunction in depression in the context of dementia prevention. Theme one explored how cognitive dysfunction is conceptualised and understood. Theme two explored potential explanations for why cognitive dysfunction may persist after depression symptoms have lifted and theme three encompassed depression and dementia risk, including risk communication practices.

### Master theme 1: cognitive dysfunction in depression

The interviews detailed how HSCPs defined cognitive dysfunction in depression, their views on why it may persist after mood symptoms have lifted and current practices around its screening.

#### Defining cognitive dysfunction in depression

Across participants, cognitive dysfunction in depression was described as a multifaceted breakdown in mental processing, centring on impairments in attention, memory and executive function. Terms such as ‘brain fog’ were used to describe individuals who struggled to concentrate, attend to information and encode memories, leading to noticeable deficits in recall and everyday functioning.

Kind of like brain fog experience and finding things difficult, finding it difficult to concentrate, memory problems. So somebody struggling to kind of concentrate, attend to information. It’s going to be very difficult for somebody to kind of encode that information and therefore kind of retrieve it and remember it later. If we think about higher level executive functioning and abilities that I think when people are struggling with depression or like a depression and anxiety mix, things like decision making and kind of planning organising can feel very overwhelming and very difficult for people to do when they’re experiencing depression particularly. (3-Clinical psychologist)

HSCPs also highlighted how rumination diverted cognitive resources, leading to divided attention and forgetfulness.

Cognitive dysfunction is forgetting things, I think it’s maybe because of divided attention because you’re ruminating on something, putting all your attention in one place. (9- Psychiatrist)

Executive functioning was consistently reported by HSCPs as being affected as part of cognitive dysfunction in depression, especially skills such as decision making, planning and organisation. These higher-order functions were reported as becoming overwhelming during depressive episodes.

Cognitive dysfunction you know in terms of your executive functioning being impacted. So you can’t actually process things, including feelings emotions (4-Psychologist)

In addition to this, HSCPs from medical backgrounds drew on neurobiological mechanisms to explain these dysfunctions, pointing to interactions between the limbic system and prefrontal cortex. It is suggested that depression may lead to limbic dominance, where emotional processing overrides the executive control normally exerted by the prefrontal cortex.

There is a lot of interaction between the limbic system and the prefrontal cortex which is responsible for executive functioning. So I feel that when people are depressed, there a lot of the decision making, a lot of the thinking involves the limbic system and they’re unable to use the prefrontal cortex. (12-Junior doctor)

#### Screening for cognitive dysfunction in depression

Participants reported that cognitive dysfunction was not routinely assessed in cases of depression. Instead, it was typically regarded as part of the overall depression symptomology rather than a primary focus. This is mirrored in the range of screening instruments often used in secondary or primary care, which often only include a few (if any) questions that might support the identification of ongoing cognitive symptoms.

I wouldn’t say it’s something we focus on, we class it as a symptom of depression, but it’s not necessarily focused on in the sessions really maybe like as a byproduct. It’s not something we measure separately, I mean just the question that’s on the PHQ-9 and the exact question is the one that I think is relevant to what we’re talking about is, ‘trouble concentrating’. (1-Cognitive behavioural therapist)

It’s not a routine thing to assess cognitive dysfunction in depression, only when that kind of question comes usually around kind of differentiating between depression and dementia. Could it be that it is a mixed picture with dementia process or could it be that we’ve kind of missed dementia. So we quite frequently use a screening tool called the ACE, so the Addenbrooke Cognitive Examination, and it’s a screening tool. It’s not a diagnostic tool, but it’s often used to support diagnosis and so it’s gives a kind of a quick overview over short term memory and language difficulties, visual spatial and difficulties and it’s done within kind of 20 min, half an hour, and it’s definitely something that kind of helps that little bit. (2-Clinical psychologist)

Specific screening tools for cognition were reported such as the Addenbrooke’s Cognitive Examination (ACE), MoCA (Montreal Cognitive Assessment) and RUDAS (Rowland Universal Dementia Assessment Scale).

I do MoCA, Addenbrookes, RUDAS. I also do PHQ-9 and GAD-7 as it gives me a better understanding overall of their cognition and their mood. There may be dementia actually as a consequence, there is also depression and anxiety, you know, which is challenging to differentiate and also be able to treat at the same time. (6-Neurologist)

Participants acknowledged that tools to screen for cognition in dementia may not be appropriate for screening cognitive dysfunction in depression, and mood only screening measures such as the PHQ-9 or HADS (Hospital Anxiety and Depression Scale) do not adequately capture cognitive symptoms.

The cognitive tests we use for dementia are not useful for depression. The one I know of that people used to assess depression, HADS, I don’t think it asks too much about cognition. I can’t see anything about memory. (9-Psychiatrist)

I do PHQ-9, which is standardised screening measure. There’s not much around memory and cognition which is important to capture. But I think it’s a very important part of a formulation and I think it’s more person centred to be able to say, well you know, are you forgetting things? Are you remembering things or how you’re thinking, you seem to get stuck. (4: Psychologist)

### Master theme 2: persistence of cognitive dysfunction

HSCPs offered a nuanced and multifactorial view of what contributes to persistent cognitive dysfunction even when mood symptoms have lifted.

#### Neurological explanations

Prolonged cognitive dysfunction was attributed to underlying neurological or medical comorbidities, such as stroke.

I’m not completely sure. It’s a bit of a mixed picture, so when I have come across this there has often been a history of strokes. So that some of those kind of symptoms are also explainable by neurological differences in their brain and then that makes kind of a little bit more sense of why they’re persisting after depression is lifting. (2- Clinical psychologist)

#### Substance abuse

The role of substance use, especially alcohol and drugs, in worsening or prolonging cognitive dysfunction was highlighted across participants. It was detailed that combining prescribed medication for depression with alcohol or cannabis may counteract therapeutic effects and exacerbate cognitive deficits.

I think cognitive dysfunction is worse in people who drink heavily or used other substances. (11-Mental health nurse therapist)

Drug and alcohol abuse, I do believe that’s had a massive impact. (3-Clinical psychologist)

Someone was taking drugs and alcohol as well another one. So if they are taking the mental health medication but also taking alcohol or cannabis or something on the side that’s actually going to make it worse. (7-Occupational therapist)

#### Role of education

A person’s educational background was perceived to influence the severity of cognitive dysfunction, indicating that higher levels of education might be associated with cognitive resilience. Similarly, lack of cognitive stimulation, particularly post-therapy, was suggested as a potential reason why cognitive dysfunction may persist beyond the resolution of mood symptoms.

Yeah, I think I’ve noticed most people who are like from a better educational background, they seem more cognitively aware during the depression than other people. So I think that might be one of the contributing factors. That could exacerbate cognitive impairment during depression. (11-Mental health nurse therapist)

If they’re not working on that cognitive stimulation and reprogramming the brain, then those deficits will surely stay there. So, although the depressions gone, the risk factor for deficit later down the line is still there because the repair hasn’t been made. (5-Psychotherapist)

#### Depression: prodrome to dementia

Uncertainty was also expressed around the boundaries between depression-related cognitive dysfunction and early neurodegenerative conditions, particularly in the older population. This points to diagnostic ambiguity that may shape how professionals interpret persistent cognitive dysfunction, blurring the lines between psychiatric and neurological explanations.

Is it depression or sort of could be a precursor to dementia. (3-Clinical psychologist)

It could be like early stages of dementia or mild cognitive impairment. (8-General Practitioner)

### Master theme 3: depression and dementia risk

This theme explored HSCPs’ views around depression as a potentially modifiable risk factor for dementia, including risk communication in health and clinical settings.

#### Views on depression as a risk factor

HSCPS generally acknowledged a plausible link between depression and increased risk of developing dementia, though the extent and nature of this relationship remained a topic of cautious interpretation. It was suggested by HSCPs that there was not enough evidence on the topic, indicating a need for further research.

Dementia has a lot of risk factors, so I think depression is very plausible. (12-Junior doctor)

Prognostically I would struggle to say that the people I’ve seen with depression and some cognitive symptoms are more likely to develop dementia. It’s completely unclear what causes what and I would question those kind of studies, it could be the other way around. But also we know that some kind of psychiatric disorders can be triggered factors or can be contributing factors to dementia. (3- Clinical psychologist)

Untreated or recurrent depression was highlighted as a contributor to long-term deterioration in brain health, potentially elevating the risk of dementia. This perspective was underpinned by observations that depressive episodes, particularly when unaddressed, may lead to behavioural and neurological changes that compromise cognitive resilience over time.

Those people who did seek support might be less likelihood of it kind of impacting their brain health. But those that didn’t might be at higher risk of developing dementia. (1-Cognitive behaviour therapist)

Depression itself is a risk factor, and if it’s left untreated, it’s more of a risk factor and. Back in the days, they used to say that the antidepressants were a risk factor to develop dementia. But now the newer studies have proved that that’s not true and in fact, treating depression should be a priority as to avoid any further deterioration in brain health. (9-Psychiatrist)

The kind of behavioural changes that are associated with depression can act as risk factors for brain health and that might in turn mean we’re more likely to experience or be diagnosed with dementia. (4-Clinical psychologist)

HSCPs also cited anecdotal experiences where patients initially diagnosed with depression later developed dementia.

I think definitely and that’s why it is important to try and treat the depression. I’ve definitely seen some cases where the person has been depressed and they get treated for depression and they do end up being again diagnosed with dementia like 2 years later. So I’ve seen actually quite a few times. (8-General practitioner)

#### Risk communication

The potential link between depression and dementia risk or ‘brain health’ was not explicitly communicated to service users. Instead, the importance of social connectedness and well-being was recognised for their protective role against depression.

I’m not necessarily always talking about it in terms of brain health per say, but maybe making links with physical health and mental health. I don’t specifically talk about it as brain health or highlight that as a potential risk factor. Good question and something I’ll definitely think about a bit more. (3-Clinical psychologist)

And we know that for every social group that you’re connected with above two, then you are protect against depression, but also, you’re improving your connectedness and well-being. But we won’t really talk about the brain. I don’t talk about brain health. (11-Mental health nurse therapist)

While links between physical and mental health were sometimes drawn, participants reported that they rarely framed these discussions explicitly in terms of brain health. Instead, the focus remained on physical health such as linking diabetes with increased risk of heart disease.

I think we’re a little bit behind. A lot of it is about diabetes and cholesterol, but really people focus on managing those to help with the heart. There’s less of a conversation around those same modifying things helping with the brain. (8-General Practitioner)

I don’t think we’ve ever told a patient like diabetes can increase the risk of dementia. We always tell them that diabetes will increase the risk of heart disease, myocardial infarction. But we should actually start telling people about other things like their brain as well, because it’s as important as physical health. (12-Junior doctor)

Due to the nature of mental health issues, in some cases, service users may reach out during crisis points. Thus, the priority was to address immediate risk such as suicidal ideation.

We usually try and solve dangerous risks for the patient or other people, like any suicidal ideation. (12-Junior doctor)

There is a great importance on the riskier aspects of suicidal ideation. (4-Psychologist)

Participants reported that conversations around brain health may be constrained by stigma and the psychological impact of diagnostic labels, where both clinicians and service users avoid engaging with the topic out of fear or discomfort.

There’s a lack of priority in going into that. There’s a fear factor that once I’m given a possible label, I will become that label. (10-Senior PWP)

## Discussion

This study explored how HSCPs conceptualise and assess cognitive dysfunction in depression and how they understand and communicate its potential role in brain health and dementia risk. Three overarching themes were developed: (1) Cognitive dysfunction in depression, (2) Persistence of cognitive dysfunction and (3) Depression and dementia risk.

Cognitive dysfunction in depression was conceptualised as difficulties occurring in concentration, memory, attention and executive functioning. This is in line with existing studies. For example, in a survey study, clinicians highly agreed that cognitive dysfunction in depression impacted these areas.^11^ Qualitative studies have also reported on cognitive dysfunction in depression having a negative impact on memory/attention/concentration and the impact of this on psychosocial and occupational functioning.^2^ In addition to this, a number of meta-analyses confirm deficits in executive function even after depression symptoms have lifted, which may explain persistent psychosocial challenges despite apparent clinical recovery.^14 15^

Due to a lack of evidence base and clinical guidelines, cognitive dysfunction in depression is not routinely screened for currently. If HSCPs did have concerns regarding a service user’s cognitive functioning during depression, then tools, such as MoCA, RUDAS and ACE were used. However, these tools are not specifically designed for screening subjective or objective cognitive dysfunction in depression. Similar findings were reported in a review on cognitive dysfunction in MDD and bipolar disorder.^16^ Specific screening measures and tools are available, including subjective measures such as the Perceived Deficits Questionnaire-Depression (PDQ-D).^17^ The PDQ-D is a 20-item self-reported, subjective measure covering the cognitive domains of attention, concentration, retrospective memory, prospective memory, planning and organisation. Studies have validated the PDQ-D in various ethnic groups and have found it to be a reliable measure to assess subjective cognitive dysfunction in depression.^18^,^20^ The THINC-it tool is another promising screening measure, developed by McIntyre *et al*,^21^ which combines subjective and objective measures. The tool was found to be valid and sensitive for use in adults with depression and was reported as quick and easy to use.

Despite promising research in this area, further work is required to understand how to respond to either subjective or objective cognitive dysfunction test scores. For example, screening of cognitive functioning in depression may support how treatment plans are shaped, with a focus on functional recovery. Similarly, it can be difficult to assess objective cognitive dysfunction in depression, which has been shown to be relatively subtle and influenced by factors such as age of onset, severity and number of episodes.^7^ Further validation studies of subjective and objective cognitive functioning in depression are required, with a particular focus on the influencing factors discussed. Mixed-methods research is also warranted to understand how these tests will work in practice and what happens next based on outcomes of tests.

The current study adds to the literature by highlighting lifestyle factors which HSCPs reported as potentially contributing to the persistence of cognitive dysfunction such as substance use, lower levels of education and lack of activities that promote cognitive stimulation post-treatment. These findings are consistent with previous research indicating that cognitive recovery following depression may be influenced by psychosocial and behavioural variables.^22^ For example, cognitive reserve, often shaped by educational attainment and cognitively stimulating activity, has been found to buffer against neurocognitive decline in mood disorders.^23^ Similarly, substance use has been associated with cognitive symptoms impacting real world functioning but does not always translate to objective cognitive dysfunction.^24^ Studies exploring cognitive symptoms in depression have highlighted a negative impact on psychosocial and occupational functioning, even after mood symptoms have lifted, causing significant distress and burden for individuals.^2^ Impairment in cognitive functioning has been shown to contribute towards the maintenance of depressive symptomology, reduced daily functioning and recurrence of depression.^25^ Recurrent depression across the life course has been associated with increased risk of suicide, hospitalisation and the development of long-term conditions such as dementia.^6 26 27^ The findings of this study support a need for further research into interventions targeting cognitive functioning in mood disorders. Cognitive remediation therapy (CRT) is a promising treatment, while most of the evidence base to date is on schizophrenia, some research on depression has found an improvement in cognitive functioning, with small significant effects on depressive symptoms and subjective daily functioning.^28^ However, follow-up and longitudinal studies are lacking to understand whether CRT in depression leads to transfer/durable effects post-treatment, indicating a gap for future research.

Our study found that despite some awareness of depression as a dementia risk factor, HSCPs rarely communicated this to service users. Conversations around brain health were uncommon, and when health risk was discussed, it focused on more established physical links, such as between diabetes and cardiovascular disease. This selective framing reflects a broader invisibility of mental health within public narratives of dementia prevention.^3^ The current findings highlighted practical and ethical challenges to discussing dementia risk. These included the need to prioritise acute concerns (eg, suicide risk), the fear of reinforcing stigma, and the worry that diagnostic labels might induce anxiety in patients. These concerns echo wider debates around the ethics of dementia risk communication.^29^

Nonetheless, HSCPs acknowledged the potential value of reframing discussions around depression potentially being a risk factor for poorer brain health and integrating this into psychoeducation or relapse prevention strategies. This could involve exploring protective behaviours, such as cognitive stimulation, social engagement and lifestyle modification in a way that empowers service users without evoking fear. Adopting a strengths-based, future-oriented approach to brain health may help bridge current communication gaps while supporting early prevention.

### Strengths and limitations

The sample of this study was diverse in terms of professional backgrounds represented and geographically. The themes represented views across HSCPs within different roles and services, which strengthens the relevance and transferability of the findings for multidisciplinary teams working in mental healthcare. However, it must be noted that some professionals were more represented than others. For example, half of the sample was from a psychological professional background, which may have influenced some of the views highlighted in this paper. The use of a purposive and convenience approach to sampling may limit the generalisability of findings. For example, some participants were recruited through ageing or dementia-related events, potentially compromising external validity of the study findings.

### Conclusion

The findings of this study suggest further research is required to understand how best to tackle cognitive dysfunction in depression and post-depression, including further testing of screening measures of cognitive dysfunction in depression. Improved communication regarding the association between depression and potential dementia risk is also warranted. Framing such discussions in terms of brain health may facilitate more constructive patient engagement and support behaviour change. To enable these conversations, further research is required to understand what type of guidance is required and the feasibility of this being integrated into mental health services. Concurrently, further research is needed to develop and evaluate interventions aimed at improving cognitive functioning in depression, with a focus on functional as well as symptomatic recovery.

## Supplementary material

10.1136/bmjopen-2025-109285 online supplemental file 1

## Data Availability

Data are available upon reasonable request.
