# Supplementary material for: Understanding cognitive dysfunction in depression: perspectives and practices of UK health and social care professionals, a qualitative study
Source: BMJ Open. 2026 Feb 12;16(2):e109285. doi: 10.1136/bmjopen-2025-109285 (PMC12911808; doi:10.1136/bmjopen-2025-109285)
Supplement: online supplemental file 1 [file bmjopen-16-2-s001.pdf]

Interview guide

Health and Social Care professionals

Health professionals

1. **How much knowledge do you currently have around cognitive dysfunction (CD) in depression?**
   Prompt: what are the symptoms?
   How prevalent is it?

2. **Do you currently assess CD in depression? If yes, how? If no, why?**
   Prompt: barriers to assessment: measures for CD in depression

3. **How confident do you feel in working with depressed individuals affected by cognitive dysfunction?**
   Prompt: is CD incorporated into treatment approaches?
   Do you think it's an important outcome to manage/treat?
   What do you think is the most effective treatment?
   What are the challenges of addressing CD in depression?

4. **What are your thoughts on symptoms of cognitive dysfunction persisting after remission of depression?**
   Prompt: do you agree/disagree with this? Explain why
   What is the impact of this?

5. **How do you think CD in depression impacts a person's day to day life?**
   Prompt: working life, social life, relationships, self-confidence?

6. **What are your views around depression and CD being a risk factor for future memory issues/dementia?**
   Prompt: do you think there is a link?
   How can we reduce risk?
