# Supplementary material for: Understanding cognitive dysfunction in depression: perspectives and practices of UK health and social care professionals, a qualitative study
Source: BMJ Open. 2026 Feb 12;16(2):e109285. doi: 10.1136/bmjopen-2025-109285 (PMC12911808; doi:10.1136/bmjopen-2025-109285)
Supplement: Supplementary file 2 [file bmjopen-2025-109285.reviewer_comments.pdf]

PEER REVIEW HISTORY

BMJ Open publishes all reviews undertaken for accepted manuscripts. Reviewers are asked to complete a checklist review form and are provided with free text boxes to elaborate on their assessment. These free text comments are reproduced below.

ARTICLE DETAILS

Title (Provisional)

Understanding Cognitive Dysfunction in Depression: Perspectives and Practices of UK Health and Social Care Professionals, a qualitative study.

Authors

Akhtar, Amirah; Nwofe, Emmanuel; Shafiq, Shabana; Parveen , Sahdia; Windle, Karen

---

VERSION 1 - REVIEW

**Reviewer**	**1**
**Name**	**Farooq, Saeed**
**Affiliation**	**Keele University, Psychiatry**
**Date**	**23-Oct-2025**
**COI**	**None**

This study aims to explore how health and social care professionals (HSCPs) in the UK conceptualise and respond to cognitive dysfunction in depression. This is important area and has not been adequately investigated from a HCP perspective. A mixed method approach would have been ideal but given the objectives of the study, present methodology is reasonable. The sample size is small and diversity in the sample could add valuable findings. It would be interesting, for example, to see different perspectives from mental health, primary care, and social care professionals. One added value of this paper lies in connecting cognitive dysfunction and related practices or experiences to dementia-risk communication and “brain health” discourse. These aspects should be more emphasized in the study's rationale. I would like authors to address following:

• Sample size (n=12) is justifiable for thematic saturation but not discussed or demonstrated.

• Data analysis: Thematic analysis is used, but it’s not specified whether it followed Braun & Clarke’s reflexive or codebook approach. A brief explanation of the analysis would be helpful for transparency.

• Results: the “persistence of cognitive dysfunction” could serve as a separate Master-theme due to its critical information.

• The findings underplay variations between disciplines, experience levels, and care settings, resulting in loss of potentially rich comparative insights.

• The results section should solely present the findings without discussing them. In nearly every theme or subtheme, there is the author's commentary. For example, "These findings emphasize how emotional and cognitive processing are interwoven and mutually affected." Similarly, "This suggests an implicit link to cognitive reserve theory, where baseline intellectual engagement may buffer against cognitive decline." These are typical discussion points.

• The discussion reads more like a policy briefing than a critical interpretation; reflexivity and theoretical depth are minimal. Some points labeled as "results," such as the examples mentioned above, could be better discussed in the discussion section.

**Reviewer**	**2**
**Name**	**Denno, Peter**
**Affiliation**	**Imperial College London**
**Date**	**31-Oct-2025**
**COI**	**None**

It was a pleasure to review this paper reporting an exploratory qualitative study of health professionals' understanding of cognitive dysfunction in depression which raises some interesting questions. The topic is novel and development in subsequent research might inform guidance for professionals or policymakers. Overall I think this could be suitable for publication following major revisions. I include specific comments below.

- Sample. Convenience and snowball sampling was used to gather a sample of 12 health professionals. A wide range of clinicians were recruited, which is a strength, however, some professions are represented by only one participant. Over-representation of psychologists in the sample is appropriately addressed in the limitations. However, the use of a convenience-type sampling approach and how this shapes the results not addressed in the limitations. Further, the paper does not discuss whether qualitative data saturation was reached, and how the sampling process may have affected this.

- Qualitative approach. There is no mention of the qualitiative approach for data analysis and philosophical assumptions used. Fupdatheremore there is no discussion of researher characteristics and reflexivity (preconceptions). These limit interpretability of the study.

- Lived experience involvement. Involvement of people with lived experience in the study development is a strength of the study. However, the nature and influence of this is not reported, could this be clarified?

- "Across HSCPS, there was a clear pattern of under-communication around brain health, particularly in the context of depression as a potentially modifiable risk factor for dementia." "Guidance is required for HSCPS to communicate depression as a risk factor for brain health."

This may be overstated or overconcluded based on the data - further studies would be required to justify guidance.

I think the the paper would be strengthened by a clearer statement and justification of authours' assumptinons / conception of 'brain health' and potentially approaching this in a more critical way. Brain health is a very broad term - what definition is being used here? Is it referring to dementia risk, or something broader? At points the interview questions and reporting imply that the authours see 'brain health' as a useful and less threatening term or concept to communicate dementia risk - is this the case? If so, it should be stated and justified.

Language should also be clearer around this - what does it mean for depression to be a 'risk factor for brain health' - is depression a risk factor for better or worse brain health? or is it an aspect of brain health?

- Cognitive dysfunction. Cognitive dysfunction is discussed in the introduction without differentiation between subjective cognitive symptoms and deficits in cognition measured by tests. However, these are dissociable and for clarity should not be conflated - many studies find they have little or no correlation (Cuevas 2022; Self-Reported Cognitive Function in Persons with Nonneurological Chronic Diseases: A Systematic Review; J Aging Research). Several studies find that negative subjective evaluations of cognition / cognitive symptoms are associated with low self-esteem and low self-efficacy, and expectancy effects (Teodoro 2018. A unifying theory for cognitive abnormalities in functional neurological disorders, fibromyalgia and chronic fatigue syndrome: systematic review. JNNP).

- "Interventions that target both depressive symptoms and cognition are required, particularly for minority ethnic communities who have shown to be at greater risk of multiple potentially modifiable risk factors for dementia." and "Experiences of HCSPs in the UK suggest a clear need for enhanced training and support in the identification and management of cognitive dysfunction associated with depression. Addressing this need requires the implementation of those validated cognitive screening tools that are feasible for use in routine clinical and therapeutic settings"

These statements may be over-concluding from the evidence presented. The results could justify further research into interventions which target objective cognition and cognitive symptoms associated with depression (for example, feasibility, acceptability, and efficacy). Likewise, the use of screening questionnaires would need further justification (what would these screen for? How would HCPs and patients respond to a positive or negative result on screening tools for subjective or objective cognitive dysfunction? What kind of studies would be needed to guide this?). Using objective cognitive tests to screen for cognitive problems in

depression could be difficult, as the deficits in cognition found in depression are relatively subtle compared to overall population variance (as per referenced Semkova meta-analysis) so may not be detected via screening tools. Furthermore, cognitive dysfunction detectable on tests should probably prompt investigation of alternative causes.

Language

- The standard of English is generally good, however, I would suggest careful copy editing and reference to journal style guide - e.g. do you need to include authours' first names in references?

- An interview transcript includes "mocha" - should this be "MoCA"?

VERSION 1 - AUTHOR RESPONSE

**Reviewer 1 comments**	**Author response**
Sample size (n=12) is justifiable for thematic saturation but not discussed or demonstrated.	We have now added in more detail around the data saturation process.
Data analysis: Thematic analysis is used, but it's not specified whether it followed Braun & Clarke's reflexive or codebook approach. A brief explanation of the analysis would be helpful for transparency.	We have now added information about the approach to thematic analysis (codebook) and further details around the process of analysis.
Results: the "persistence of cognitive dysfunction" could serve as a separate Master-theme due to its critical information.<br><br>The findings underplay variations between disciplines, experience levels, and care settings, resulting in loss of potentially rich comparative insights.	Thank you for highlighting this, we have now made this into a separate theme.<br><br>Thank you for your comment. We have now added this point as a limitation of the study.
The results section should solely present the findings without discussing them. In nearly every theme or subtheme, there is the author's commentary. For example, "These findings emphasize how emotional and cognitive processing are interwoven and mutually affected." Similarly, "This suggests an implicit link to cognitive reserve theory, where baseline intellectual engagement may buffer against cognitive decline." These are typical discussion points.	We have now removed points of discussion from the results section and moved these to the discussion section.
The discussion reads more like a policy briefing than a critical interpretation; reflexivity and theoretical depth are minimal. Some points labelled as "results," such as the examples mentioned above, could be better discussed in the discussion section.	We have updated the discussion section and removed points to avoid it reading like a policy briefing. By moving the discussion presented originally in the findings section to the discussion section, the discussion now presents more critical interpretation.

**Reviewer 2 comments**	**Author response**
**Sample.** The use of a convenience-type sampling approach and how this shapes the results not addressed in the limitations.<br><br>Further, the paper does not discuss whether qualitative data saturation was reached, and how the sampling process may have affected this.	We have now added this to the strength and limitations section.<br><br>We have now added in more detail around the data saturation process, making reference to the diversity of the sample.
**Qualitative approach.** There is no mention of the qualitative approach for data analysis and philosophical assumptions used.<br><br>Furthermore, there is no discussion of researcher characteristics and reflexivity (preconceptions). These limit interpretability of the study.	The approach to data analysis (codebook thematic analysis) and philosophical assumptions (critical realism) has now been added to the methods section.<br><br>We have now added a section on research reflexivity and characteristics for all authors.
**Lived experience involvement.** Involvement of people with lived experience in the study development is a strength of the study. However, the nature and influence of this is not reported, could this be clarified?	We have added further details regarding how PPI shaped the design of this study.
- *"Across HSCPS, there was a clear pattern of under-communication around brain health, particularly in the context of depression as a potentially modifiable risk factor for dementia." "Guidance is required for HSCPS to communicate depression as a risk factor for brain health."*<br>**This may be overstated or over concluded based on the data - further studies would be required to justify guidance.**	We have now amended this statement in the abstract.
I think the paper would be strengthened by a clearer statement and justification of authors' assumptions / conception of 'brain health' and potentially approaching this in a more critical way. Brain health is a very broad term - what definition is being used here? Is it referring to dementia risk, or something broader? At points the interview questions and reporting imply that the authors see 'brain health' as a useful and less threatening term or concept to communicate dementia risk - is this the case? If so, it should be stated and justified.	In the introduction where the term 'brain health' is first used, we have expanded on what this means for the context of this paper. We have explained that using the term brain health may resonate more with the public, as opposed to dementia and therefore may lead to behaviour change. We have supported use of this term in this context by referencing dementia charities utilising the concept of brain health.
Language should also be clearer around this - what does it mean for depression to be a 'risk factor for brain health' - is depression a risk factor for better or worse brain health? or is it an aspect of brain health?	We have clarified the meaning of brain health in the context of depression and dementia risk – with emphasis on depression as

	potentially being a risk factor for poorer brain health.
**Cognitive dysfunction.** Cognitive dysfunction is discussed in the introduction without differentiation between subjective cognitive symptoms and deficits in cognition measured by tests. However, these are dissociable and for clarity should not be conflated - many studies find they have little or no correlation ***(Cuevas 2022; Self-Reported Cognitive Function in Persons with Nonneurological Chronic Diseases: A Systematic Review; J Aging Research).***<br>Several studies find that negative subjective evaluations of cognition / cognitive symptoms are associated with low self-esteem and low self-efficacy, and expectancy effects ***(Teodoro 2018. A unifying theory for cognitive abnormalities in functional neurological disorders, fibromyalgia and chronic fatigue syndrome: systematic review. JNNP).***	We have now discussed the difference between subjective and objective cognitive dysfunction. We have added in further studies that demonstrate an association between subjective cognitive dysfunction (SCD) and risk of objective cognitive disorders/mild cognitive impairment/dementia. Whilst highlighting that having SCD does not mean you will go onto develop objective cognitive disorders. We have strengthened the rationale for conducting further research on SCD. We have also updated the aims and research question to specify our focus on SCD.
- *"Interventions that target both depressive symptoms and cognition are required, particularly for minority ethnic communities who have shown to be at greater risk of multiple potentially modifiable risk factors for dementia."*<br>and<br>*"Experiences of HCSPs in the UK suggest a clear need for enhanced training and support in the identification and management of cognitive dysfunction associated with depression. Addressing this need requires the implementation of those validated cognitive screening tools that are feasible for use in routine clinical and therapeutic settings"*<br><br>These statements may be over-concluding from the evidence presented.	The first statement was in the abstract, since re-structuring of the abstract, this statement has been removed.<br><br>The second statement from the conclusions section has been re-worded to avoid over-conclusions from the evidence presented.
The results could justify further research into interventions which target objective cognition and cognitive symptoms associated with depression (for example, feasibility, acceptability, and efficacy).<br><br>Likewise, the use of screening questionnaires would need further justification (what would these screen for? How would HCPs and patients respond to a positive or negative result on screening tools for subjective or objective cognitive dysfunction? What kind of studies would be needed to guide this?). Using objective cognitive tests to screen for cognitive problems in depression could be difficult, as the deficits in cognition found in depression are relatively subtle compared to overall population variance (as	We have now added in discussion around future research into interventions, focusing on cognitive remediation therapy as this is one of the well-established interventions for cognitive functioning in mental health conditions.<br><br>Screening questionnaires: we have re-phrased this section to avoid any over-conclusions. We have now discussed the complex nature of screening Subjective and objective cognitive dysfunction and the many unanswered questions regarding implementation of screening into practice.

per referenced Semkova meta-analysis) so may not be detected via screening tools. Furthermore, cognitive dysfunction detectable on tests should probably prompt investigation of alternative causes.	We have justified a need for further research in this area and given examples.
**Language**<br>The standard of English is generally good, however, I would suggest careful copy editing and reference to journal style guide - e.g. do you need to include authours' first names in references?<br><br>An interview transcript includes "mocha" - should this be "MoCA"?	Apologies this was an issue with Endnote – citations in text have now been updated.<br><br>Thank you for highlighting, the spelling has now been amended to MoCA.

VERSION 2 - REVIEW

Reviewer 2

Name Denno, Peter

Affiliation Imperial College London

Date 22-Dec-2025

COI

The revised manuscript is improved and gives a clearer presentation of the study, particularly the background, methods and results. In my view, the manuscript could be suitable for publication following minor revisions.

There are some remaining statements which are not supported by the study findings. For example, in the abstract, the conclusion states, "SCD in depression is important to address to improve individuals' psychosocial and occupation functioning, to avoid further episodes of depression and potential deterioration in brain health." As this study investigated HSCPs views and understanding, findings about this should be highlighted.

Some statements the results section still add interpretation or imply generalisability. For example, "Across HSCPS, there was a clear pattern of under-communication around brain health"; and "There was a general consensus among HSCPs". It may be better to refer to "participants".

In the revised manuscript, the term "SCD" is used throughout to refer to "Subjective Cognitive Dysfunction". Whilst this is more specific, I have two concerns:

(1) This may introduce confusion as the acronym SCD is used to refer to "subjective cognitive decline" in dementia research, conceptualised as (subjective) cognitive symptoms, particularly memory symptoms, often with concerns about dementia, but with normal results on cognitive screening instruments (doi: 10.1016/S1474-4422(19)30368-0; doi.org/10.1038/s41380-025-03021-0). An alternate term e.g., "cognitive symptoms" would be more appropriate if referring specifically to subjective cognitive symptoms, whereas "cognitive dysfunction" is appropriate to refer to both cognitive symptoms and/or dysfunction in cognitive performance as measured on tests, where these are not distinguished.

(2) The term SCD is now used in at some points in the results section where it appears participants were asked about this, however it does not appear in the topic guide. 'Cognitive dysfunction' may be more appropriate here.

I advise each use of the term is reviewed to clarify whether it is referring to unspecified cognitive dysfunction vs cognitive symptoms vs performance on cognitive tests (or screening instruments).

VERSION 2 - AUTHOR RESPONSE

Reviewer comment	Author response
There are some remaining statements which are not supported by the study findings. For example, in the abstract, the conclusion states, "SCD in depression is important to address to improve individuals' psychosocial and occupation functioning, to avoid further episodes of depression and potential deterioration in brain health." As this study investigated HSCPs views and understanding, findings about this should be highlighted.	We have now removed the statement not supported by study findings.<br><br>As per other comments to maintain consistency, we have also replaced SCD with 'cognitive dysfunction' in the results sections of the abstract to avoid confusion around acronyms commonly used in dementia research.
Some statements the results section still add interpretation or imply generalisability. For example, "Across HSCPS, there was a clear pattern of under-communication around brain health";<br>and<br><br>"There was a general consensus among HSCPs". It may be better to refer to "participants".	Thank you for highlighting this, we have now removed this statement from the results section.<br><br>We have now amended this statement.
In the revised manuscript, the term "SCD" is used throughout to refer to "Subjective Cognitive Dysfunction". Whilst this is more specific, I have two concerns:	**Introduction**<br>We have now amended terms to 'subjective cognitive symptoms' for literature reporting on subjectively reported cognitive

(1) This may introduce confusion as the acronym SCD is used to refer to "subjective cognitive decline" in dementia research, conceptualised as (subjective) cognitive symptoms, particularly memory symptoms, often with concerns about dementia, but with normal results on cognitive screening instruments (doi: 10.1016/S1474-4422(19)30368-0; doi.org/10.1038/s41380-025-03021-0).<br><br>An alternate term e.g., "cognitive symptoms" would be more appropriate if referring specifically to subjective cognitive symptoms,<br><br>whereas<br>"cognitive dysfunction" is appropriate to refer to both cognitive symptoms and/or dysfunction in cognitive performance as measured on tests, where these are not distinguished.<br><br>(2) The term SCD is now used in at some points in the results section where it appears participants were asked about this, however it does not appear in the topic guide. 'Cognitive dysfunction' may be more appropriate here.<br><br>I advise each use of the term is reviewed to clarify whether it is referring to unspecified cognitive dysfunction vs cognitive symptoms vs performance on cognitive tests (or screening instruments).	symptoms, as opposed to using the term SCD.<br><br>**Aim and research questions:**<br>We have updated terms from SCD to cognitive dysfunction to reflect its original format and consistency with terms used in topic guide.<br><br>**PPI:**<br>We have used the term 'cognitive symptoms' when referring to PPI members, as they subjectively reported cognitive symptoms related to their depression.<br><br>**Results:**<br>We have amended from SCD to cognitive dysfunction across the results section to reflect its original format and consistency with terms used in topic guide.<br><br>**Discussion:**<br>When discussing literature on subjective cognitive symptoms we have amended terms used to 'cognitive symptoms' (page 22).

VERSION 3 - REVIEW

**Reviewer**	**2**
**Name**	**Denno, Peter**
**Affiliation**	**Imperial College London**
**Date**	**16-Jan-2026**
**COI**	

The authours have addressed the points raised in my review. I commend the authours on a valuable study, and their responses during peer review, which have strengthened the reporting.
